# Titania-Supported Photocatalytic Coatings of Cu_2_O Nanoparticles Synthesized via Heterogeneous Nucleation

**DOI:** 10.1021/acsomega.5c08505

**Published:** 2026-01-20

**Authors:** Petra Demény, Borbála Tegze, Bálint Fodor, Pál Maák, János Madarász, Zsombor Pap, Dániel Zámbó, Tamás Igricz, Adél Sarolta Rácz, Norbert Nagy, Zoltán Hórvölgyi

**Affiliations:** † Department of Physical Chemistry and Materials Science, Faculty of Chemical Technology and Biotechnology, 61810 Budapest University of Technology and Economics, Műegyetem rkp. 3., Budapest H-1111, Hungary; ‡ 249916 Semilab Semiconductor Physics Laboratory Co. Ltd., Prielle Kornélia u. 2, Budapest H-1117, Hungary; § Department of Atomic Physics, Faculty of Natural Sciences, Budapest University of Technology and Economics, Műegyetem rkp. 3., Budapest H-1111, Hungary; ∥ Department of Inorganic and Analytical Chemistry, Faculty of Chemical Technology and Biotechnology, Budapest University of Technology and Economics, Műegyetem rkp. 3., Budapest H-1111, Hungary; ⊥ Institute for Technical Physics and Materials Science, 303347 HUN-REN Centre for Energy Research, Konkoly-Thege M. út 29-33., Budapest H-1121, Hungary; # Department of Organic Chemistry and Technology, Faculty of Chemical Technology and Biotechnology, Budapest University of Technology and Economics, Műegyetem rkp. 3., Budapest H-1111, Hungary

## Abstract

Mesoporous TiO_2_ sol–gel coatings with a thickness of 122 nm and a porosity of 49% were prepared by dip-coating, followed by Cu_2_O nanoparticle deposition onto the surface using a simple, one-step method: the TiO_2_ coating was immersed in the reaction mixture and Cu_2_O particles formed on the surface in a heterogeneous nucleation process. The crystallinity, size, shape, and structure of the samples were characterized by X-ray diffraction, X-ray photoelectron spectroscopy, Raman spectroscopy, atomic force microscopy, and scanning electron microscopy. Optical properties, layer thickness, and porosity were determined by UV–vis spectroscopy and spectroscopic ellipsometry. Cu_2_O nanoparticles with an oblate spheroidal shape and cubic crystal structure formed on the surface, with an average particle size of 326 nm, and the surface coverage could be controlled by the reaction time. Photoactivity of the TiO_2_/Cu_2_O coatings was studied in dye photodegradation tests under UV and visible light, using methyl orange dye as a model pollutant. The samples showed significant photoactivity; the amount of Cu_2_O particles and their surface coverage on titania played an important role. High surface coverage could be achieved in a simple one-step deposition process using heterogeneous nucleation, resulting in enhanced photoactivity under visible light, making this method suitable to produce photoactive coatings for a variety of applications, such as air and water purification.

## Graphical abstract

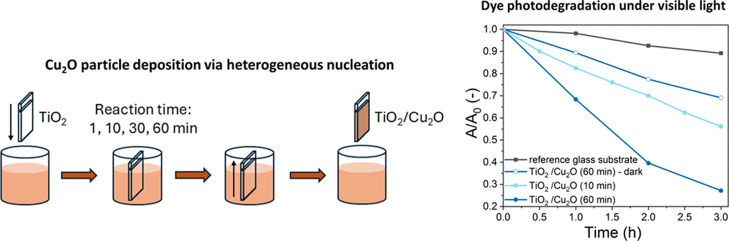

## 1 Introduction

Self-cleaning surfaces can remove adsorbed pollutants from their surface with the help of environmental factors. This property can be achieved by two main types of coating design: (1) the surface is superhydrophobic and water-repellent, the pollutants can be washed away by the water droplets (e.g., rain drops);^1^ (2) the surface has photocatalytic activity made of a semiconductor generating photoexcited charge carriers by electromagnetic radiation (e.g. sunlight) and the electrons migrate from the valence band to the conduction band, leaving holes behind. These photoexcited charge carriers are able to degrade organic molecules through redox reactions.^2^ TiO_2_ is a semiconductor metal-oxide, which shows high photocatalytic activity but is only active under UV irradiation due to its large band gap (3.2 eV for anatase crystal structure).^3,4^ For many applications, it would be beneficial to utilize a wider wavelength range of sunlight and prepare photocatalysts that are also active under visible light. There are several solutions to achieve this, for example, modification of TiO_2_ by metals,^5^ nonmetals,^6^ or other semiconductors^7^ with smaller band gaps.^8^

The photoactivity of the TiO_2_ can be extended by preparing a composite material that contains Cu_2_O particles, which have a smaller band gap (∼2.0–2.2 eV^9^) and show photocatalytic activity under visible light irradiation. In a semiconductor heterojunction, due to the different band gaps and band edges, discontinuity occurs in the Fermi energy, which can reach its equilibrium due to the migration of electrons and holes. The movement of the charge carriers occurs in the interfacial charge region, which causes the band bending of the composite.^10^ In this special case, the electrons of the Cu_2_O are excited by absorbing a visible light photon; then, these excited electrons are able to transfer to the conduction band of the TiO_2_, leaving the holes behind in the valence band of Cu_2_O (Figure 1).^11,12^ This mechanism provides the separation of the photoexcited charge carriers and increases their lifetime, which leads to a more efficient photodegradation of organic material, in addition to the achieved widened wavelength range of photoactivity.^13–17^

**Figure 1:**
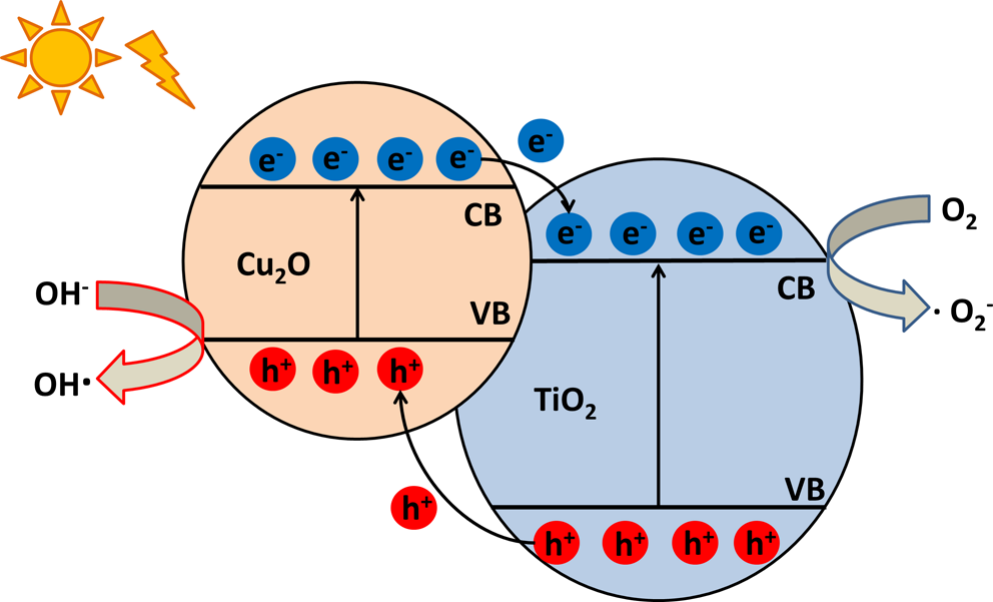
Schematic illustration of the photocatalysis process of a TiO_2_/Cu_2_O heterojunction.

Utilizing nanostructured coatings for photocatalytic air and water purification has many advantages, as evidenced by the intensive research being conducted on the topic.^18–20^ By immobilizing photocatalysts on a solid substrate and using them in coating form, their separation from the wastewater can be much more effective; their recovery and reuse are made easier, leading to a cost-effective and sustainable solution. The nanostructure (nanosized pore structure and/or coating built up of nanoparticles) ensures a high specific surface area, which is essential for the efficient photodegradation. Furthermore, as mentioned above, interactions between different nanostructured semiconductors may provide synergistic effects and lead to higher photoactivity by increasing charge separation and decreasing the recombination rate of charge carriers.^21,22^

In the literature, there is a wide range of options to obtain Cu_2_O particles deposited on a specific surface. The most commonly applied method is electrodeposition,^23^ while sputtering,^24^ laser ablation,^25^ and microsphere lithography^26^ are also used. TiO_2_/Cu_2_O composite coatings can also be achieved by heterogeneous liquid phase synthesis, by placing the TiO_2_-coated sample into a liquid reaction mixture, resulting in the formation of Cu and/or Cu_*x*_O particles on the surface. Reports can be found on heterogeneous liquid phase nucleation of Cu_2_O particles onto titania coatings achieved by hydrothermal synthesis^27^ or by immersion in a Cu­(II)-salt solution, followed by washing step and then a simple reduction step also by immersion in the solution of the reducing agent.^28,29^ From a practical point of view, simplification of the photocatalyst system preparation method can reduce costs and make the process more efficient and robust. Out of the examples mentioned in the literature for deposition of a Cu_2_O particle layer onto a titania surface, forming the particles by heterogeneous liquid phase nucleation using immersion in an aqueous solution can be regarded as the simplest method: it would be advantageous to further simplify this method by eliminating the additional steps, such as washing and immersion into other solutions and instead carrying out the Cu_2_O particle deposition in a single step.

In our study, TiO_2_/Cu_2_O composite coatings were obtained using a simplified method: the mesoporous TiO_2_ coating was immersed in the liquid reaction mixture and the Cu_2_O particles were deposited onto the surface via heterogeneous nucleation in a single step. This approach has the benefit of requiring neither special equipment nor extreme conditions, such as high temperature and pressure, while achieving the particle deposition in a single step. The mesoporous nanostructure of the TiO_2_ coatings and depositing Cu_2_O in the form of nanoparticles ensure a high specific surface area, which is essential for efficient photodegradation of organic pollutants that are adsorbed on the photocatalyst surface. The aim of our research is to study the photoactivity and other important properties (structure, morphology of nanoparticles, etc.) of the TiO_2_/Cu_2_O composite coatings prepared with this simple, one-step heterogeneous liquid phase nucleation method. The properties of the samples were characterized by X-ray diffraction, X-ray photoelectron spectroscopy, Raman spectroscopy, field emission scanning electron microscopy, atomic force microscopy, and optical spectroscopy methods, and their photoactivity was investigated based on dye photodegradation studies carried out under UV and visible light irradiation.

## 2 Experimental Section

### 2.1 Materials

Absolute ethanol (EtOH, >99,7% for analysis), 2-propanol (2-PrOH, >99,7% f. a.), sulfuric acid (H_2_SO_4_, 96%, f. a.), hydrochloric acid (HCl, 37%, f. a.), H_2_O_2_ aqueous solution (30%), sodium hydroxide (NaOH, >99%), tetraethyl orthosilicate (TEOS, >99%, f. a.), titanium­(IV), isopropoxide (TTIP, >99.7%, f. a.), acetylacetone (ACAC, >99%), copper­(II) acetate monohydrate (>98%), and methyl orange dye (>99%) were obtained from Reanal (Budapest, Hungary). Pluronic P123 (P123, PE–PP-PEO triblock copolymer, average MW = 5800 Da, >99%, f. a.) and *l* -ascorbic acid (>99%) were purchased from Sigma-Aldrich (Budapest, Hungary). Purified distilled water (filtered with Adrona Integrity + system to reach 18.2 MΩ•cm) was used in the experiments. Glass slides (76 × 26 × 1 mm, Thermo Scientific, Menzel Gläser) and silicon wafers (Si Siegert, (100) p-type, prime grade) were used as substrates for the preparation of thin coatings.

### 2.2 Preparation of Samples

TiO_2_/Cu_2_O composite coatings were prepared on glass and Si substrates. On glass substrates, first a compact silica barrier layer was deposited, followed by the deposition of a TiO_2_ layer. The role of the silica barrier layer is the inhibition of the Na^+^ diffusion from the glass into the TiO_2_ at the high temperatures used during the preparation, which could significantly decrease the photoactivity.^30^ Mesoporous SiO_2_/Cu_2_O coatings on glass substrates were also prepared for use as reference samples during the photocatalysis measurements.

Compact and mesoporous silica, and mesoporous TiO_2_ coatings were prepared by the sol–gel method. Precursor sols were synthesized by the acid-catalyzed controlled hydrolysis of metal-alkoxides in ethanol, as reported in our previous publications.^31–33^ Compact silica barrier layers were prepared from a precursor sol of TEOS:EtOH:HCl:H_2_O with molar ratios of 1:4.75:7.2 × 10^–4^:4.00. Mesoporous silica layers were made using sols containing TEOS:EtOH:HCl:H_2_O:P123 with molar ratios of 1:9.57:0.0101:5.63:0.00875. Mesoporous TiO_2_ layers were prepared using precursor sols made of TTIP:EtOH:ACAC:H_2_O:P123 with molar ratios of 1:33.80:1.93:4.39:0.034. According to our previous publications,^31,34^ transmission electron microscopy images and ellipsometric porosimetry measurements (carried out on TiO_2_ coatings prepared with the exact same methods and parameters as used in this work) showed that the mesoporous structure (average pore diameter: 9 nm) of the TiO_2_ coating results in a high specific surface area (∼1100 m^2^ cm^–3^), which leads to efficient photodegradation of dye molecules under UV irradiation. The TiO_2_ layer is built up of ∼10 nm sized TiO_2_ particles (the particle sizes change between 6 and 15 nm) with air-filled voids between them.

Glass and Si substrates were first cleaned by a detergent solution and then by 10 w/w % H_2_SO_4_ solution, 2-PrOH, and distilled water. Coating deposition from the precursor sols was carried out using a dip-coater (Plósz Mérnökiroda Kft., Hungary), at 25 °C, with a 6 cm min^–1^ withdrawal speed for the silica and 12 cm min^–1^ for the TiO_2_ coatings. The coatings were dried in air and then placed in an oven (Nabertherm B170) for heat treatment: compact silica barrier layers were treated at 450 °C for 30 min, while porous silica and TiO_2_ coatings were treated at 480 °C for 1 h.

Cu_2_O nanoparticles were synthesized and deposited onto the TiO_2_ surface using a simple, one-step deposition method (see Figure 2): the TiO_2_ coating was immersed in the reaction mixture and Cu_2_O particles formed on the surface in a heterogeneous nucleation process. The Cu_2_O particles were synthesized using the following steps: 10 mL copper­(II) acetate solution (0.1 M) and 40 mL ascorbic acid solution (0.1 M) were prepared and mixed at 35 °C and pH = 11 (pH was set by adding 2 M NaOH solution), which resulted in the formation of an orange-colored suspension of Cu_2_O nanoparticles. The reaction time was changed between 1, 10, 30, and 60 min during the TiO_2_/Cu_2_O sample preparation. The samples made with different reaction times showed different colors (for a detailed schematic of the sample preparation and for photos of the samples; see Figure S1). Reference samples of Cu_2_O particles deposited onto mesoporous silica coatings were prepared using the same steps.

**Figure 2:**
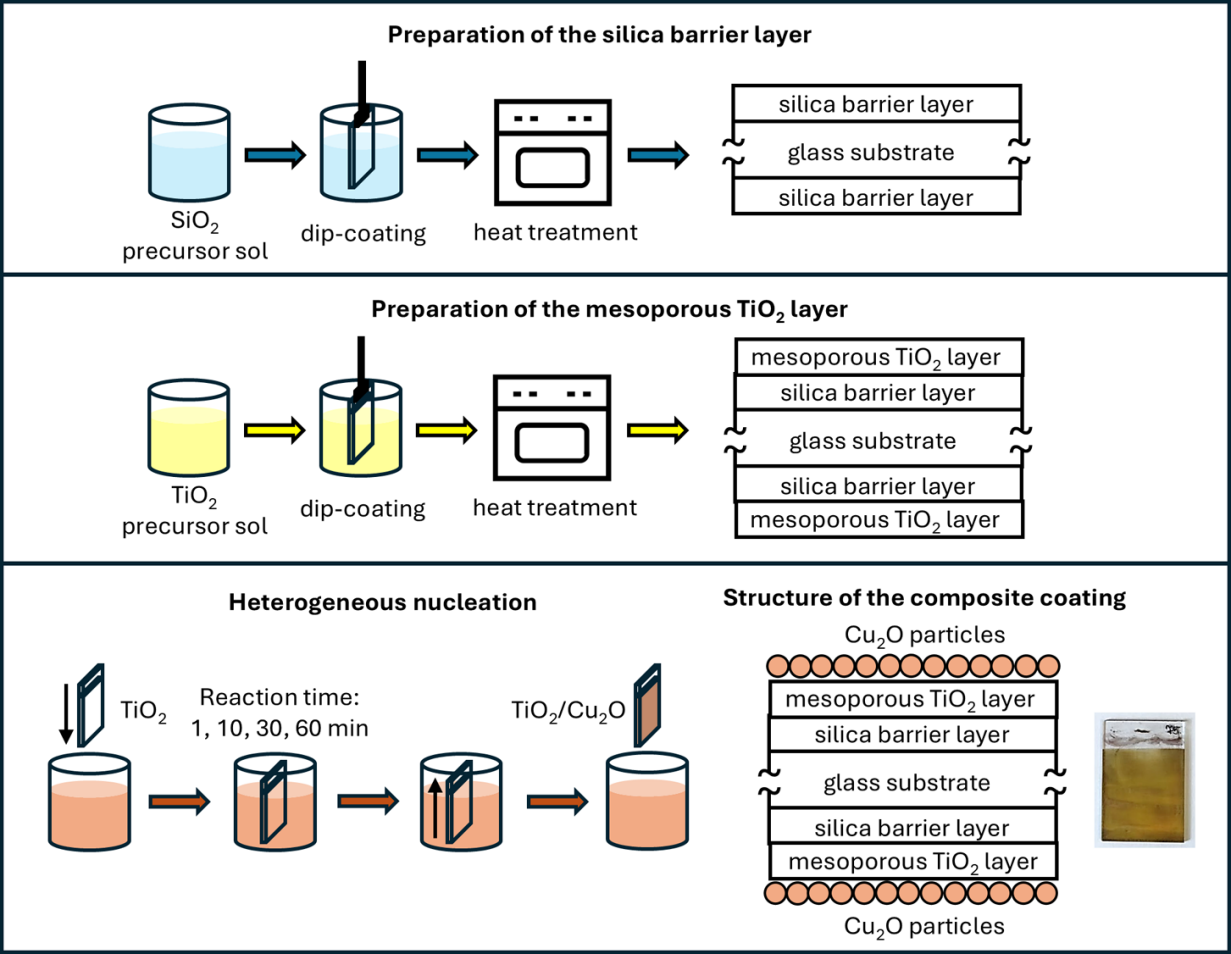
Schematic illustration of the preparation of the TiO_2_/Cu_2_O samples (the photo shows a TiO_2_/Cu_2_O coating prepared with a 60 min reaction time).

### 2.3 Characterization Methods

Optical properties of the coatings were characterized by UV–vis spectroscopy method, using an Analytic Jena Specord 200–0318 spectrophotometer: transmittance and absorbance spectra were measured in the wavelength range of 350–1100 nm, with a scanning speed of 10 nm s^–1^ and a slit width of 1 nm. The effective refractive index (at 632.8 nm) and layer thickness values of the silica and TiO_2_ coatings were determined from the transmittance data using the thin film optical model of the Hild-method.^35–37^ Spectroscopic ellipsometry was used to characterize the TiO_2_/Cu_2_O coatings made with a 60 min reaction time, using a Semilab SE-2000 ellipsometer, in the wavelength range of 250–975 nm, with three different angles of incidence values (65°, 70°, 75°). The spectra were analyzed using the Spectroscopic Ellipsometry Analyzer software; the effective refractive index, layer thickness, and band gap energy values were determined using Sellmeier and Tauc–Lorentz models.^38,39^ Porosity values were estimated based on the refractive indices using the Lorentz–Lorenz equation.^40^

The crystal structure of the Cu_2_O nanoparticles was measured on powder samples by X-ray diffraction (XRD), using a Philips PANalytical X’pert Pro device with Cu–Kα radiation, measured in the range of 2θ = 4–84°, with a 0.0167° step size and a 5 s scanning speed. The powder samples were prepared by first synthesizing the Cu_2_O particles following the same steps as above, only without immersing a TiO_2_ coating into the reaction mixture: after 60 min, the particles were separated by centrifugation (5000 rpm, 10 min, using a Hermle Z 36 K centrifuge), followed by consecutive washing steps (two times in water, one time with ethanol, always using 5000 rpm, 10 min centrifugation for separating the dry matter from the dispersion phase). Then, the particles were dried at 80 °C for 60 min, resulting in an orange-colored powder sample. The crystallite size of the Cu_2_O nanoparticles was calculated by the Scherrer equation,^41^ from the highest intensity peak observed on the XRD pattern (2θ = 36.68°, corresponding to the (111) crystal plane). The TiO_2_/Cu_2_O composite coatings were also measured, using the grazing angle mode, suitable for investigating very thin coatings, with a measurement range of 2θ = 25–50°, a step size of 0.0167°, and a scanning speed of 30 s.

Composition and structure of the TiO_2_ and TiO_2_/Cu_2_O coatings were further confirmed by Raman spectroscopy, using a Horiba Jobin Yvon LabRam 300 spectrometer equipped with a frequency-doubled Nd/YAG laser (532 nm), a Synapse Plus InGaAs detector, and an objective of 100× magnification.

X-ray photoelectron spectroscopy (XPS) measurements were carried out on a TiO_2_/Cu_2_O sample (prepared with a 60 min reaction time on silicon substrate) by a Thermo Scientific ESCALAB Xi + instrument, with an Al Kα X-ray source (λ = 0.8340 nm, 1486.6 eV), an X-ray spot size of 900 μm, and using cluster argon ion sputtering. Survey spectra were measured in steps of 0.5 eV with a 10 ms dwell time per data point. Silicon (2p), carbon (1s), copper (LMM), copper (2p), titanium (2p), oxygen (1s), sulfur (2p), and nitrogen (1s) high-resolution spectra were measured within the spectral range of interest (ca. 20 eV around the core level emission peaks) at 20 eV pass energies with 0.1 eV steps and a 50 ms dwell time per data point.

The shape and sizes of Cu_2_O particles and the structure of the coated samples were characterized by field emission scanning electron microscopy (FE-SEM), measured on TiO_2_/Cu_2_O coatings deposited onto Si substrates (prepared using the same steps and parameters as described above), using a Zeiss Leo 1540XB FE-SEM device operated at a 5 kV acceleration voltage and utilizing an Oxford Instruments UltimMax 40 detector for recording elemental maps with energy-dispersive X-ray spectroscopy (EDS).

The single-layer formation and the surface coverage of the TiO_2_/Cu_2_O composite samples were characterized by atomic force microscopy (AFM), measured on TiO_2_/Cu_2_O coatings deposited onto Si substrates (prepared with a 60 min reaction time) by an AFM device (AIST-NT SmartSPM 1000) in tapping mode with a PPP-NCHR20 needle (NanoSensors, nominal radius of the needle <20 nm). The evaluation was performed using Gwyddion software.

The photoactivity of the samples was investigated with dye photodegradation tests under UV and visible light, using methyl orange dye as a model pollutant. The coatings were placed into a crystallizing dish filled with a 25 mL 10^–5^ M aqueous dye solution. H_2_O_2_ cocatalyst (125 μL, 30% aqueous solution) was added for the visible light tests: the cocatalyst increases the rate of the degradation and decreases the required time of the measurements.^42^ In the first step, the samples were kept in darkness for 1 h (adsorption equilibrium was reached), and then they were irradiated by UV or visible light for 3 h. Absorbance spectra between 350 and 600 nm (10 nm s^–1^ scanning speed, 1 nm slit width) were taken in the beginning and after each hour of the measurement, using an optical glass cuvette and distilled water as reference. The solutions were stirred during the process, and the system was cooled by a water bath and ventilation to prevent heating by the high-intensity lamps. The irradiation sources (Life Light LED, 48 W, E27 type halogen lamp) with emission wavelengths between 400 and 750 nm and emission maximum at 595 nm was used for visible light; Phillips CLEO UV-A HPA 400 S, 400 W lamp with emission maximum at 365 nm was used for UV irradiation) were kept with their longitudinal axes perpendicular to the samples with a fixed 15 and 30 cm source–sample distance and power density of 4.37 mW cm^–2^ and 47.1 mW cm^–2^ (measured at said distances) for vis and UV irradiation, respectively.

## 3 Results and Discussion

### 3.1 Structural and Optical Properties of the TiO_2_/Cu_2_O Coatings

Figure 3a shows the XRD pattern measured on the powder sample of the Cu_2_O nanoparticles. The detected peaks belong to the cubic structure characteristic of Cu_2_O crystals (JCPDS 05-0667), while there is an additional very small peak at 52.6°, which most likely belongs to a pollutant. The average crystallite size was determined to be 21 nm, based on the highest intensity peak (111), using Scherrer’s equation.^41^ Figure 3b shows the XRD pattern of the TiO_2_/Cu_2_O coatings: due to the very thin layer of nanoparticles deposited onto the titania surface during a heterogeneous nucleation process, the grazing angle measurement mode was necessary to achieve observable peaks. Two broad peaks can be seen on the pattern at 36.7° and 42.5°, in agreement with the highest intensity (111) and (200) peaks observed on the XRD pattern of the powder samples, which suggests that the particles synthesized by heterogeneous nucleation are also made of cubic crystal-structured Cu_2_O. The results show that only Cu_2_O nanoparticles were formed on the titania surface, without the presence of crystalline Cu or CuO particles.

**Figure 3:**
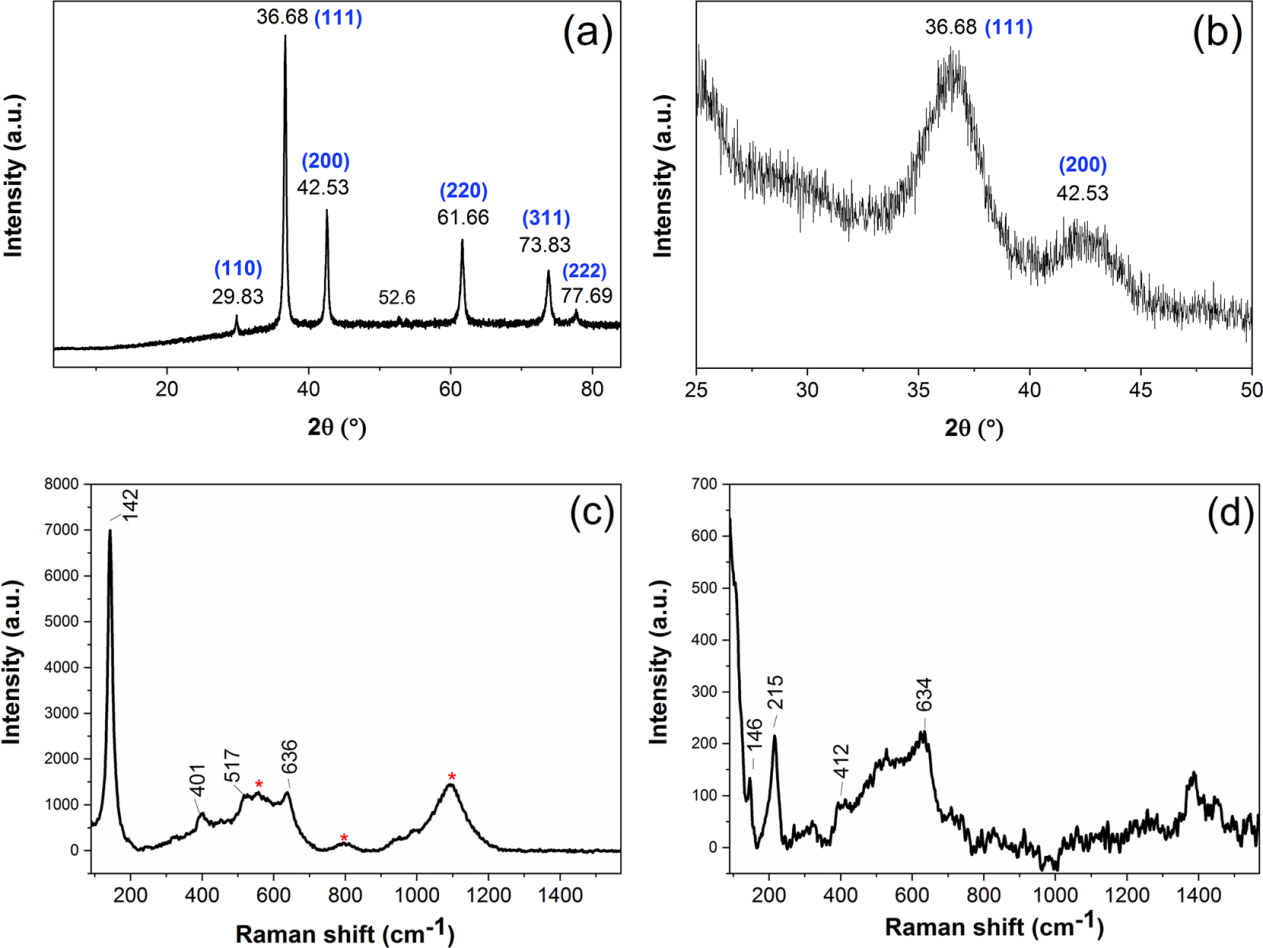
XRD and Raman results: XRD patterns of the powder sample (prepared in a homogeneous synthesis with a 60 min reaction time) (a) and the TiO_2_/Cu_2_O composite coating (prepared with a 60 min reaction time; measured using grazing angle mode) (b). Raman spectra of the TiO_2_ coating (c) and the TiO_2_/Cu_2_O coating (prepared with a 60 min reaction time) (d).

Raman spectra of the TiO_2_ and TiO_2_/Cu_2_O (prepared with a 1 h reaction time) coatings on glass were measured in order to confirm the composition and structure of the samples. The Raman spectrum of the TiO_2_ coating can be seen in Figure 3c: the bands at 142, 401, 517, and 636 cm^–1^ indicate the presence of the TiO_2_ anatase crystal structure.^43^ The bands marked with an asterisk show the presence of the glass substrate (see Figure S2 for the Raman spectrum of the reference glass substrate). Figure 3d shows the Raman spectrum of the TiO_2_/Cu_2_O coating: the bands at 146, 215, 412, and 634 cm^–1^ appear due to the Cu_2_O nanoparticles deposited onto the surface. The most distinguishable band at 215 cm^–1^ confirms the presence of Cu_2_O, as it corresponds to the second-order Raman-allowed mode 2Γ_12_^–^ of Cu_2_O.^44^ The same bands were observed on the sample prepared with a 30 min reaction time (see Figure S3) but did not appear on the spectra of the samples prepared with 1 and 10 min reaction times, likely due to the smaller amount of particles deposited onto the surface in the last two cases.

XPS measurements were carried out on the TiO_2_/Cu_2_O coating (prepared with a 60 min reaction time on silicon substrate) (see details in the Supporting Information, in Figures S4 and S5). The atomic composition of the sample was determined to be 37.8% C 1s, 23.8% Cu 2p, 30.2% O 1s, 0.9% Ti 2p, 4.0% S 2p, and 3.3% N 1s. There was no detectable amount of Si 2p. The high-resolution spectrum of the Cu 2p showed two peaks at 952.6 ± 0.1 eV and 932.5 ± 0.1 eV, which corresponds to the Cu 2p 1/2 and Cu 2p 3/2 states, respectively. With further characterization, the Auger spectrum indicated that only Cu_2_O was present in the sample.^45^

Figure 4a–c shows the FE-SEM images of the TiO_2_/Cu_2_O coatings (prepared with a 60 min reaction time), and the size distribution diagram of Cu_2_O particles can be seen in Figure 4d. It can be seen that as a result of heterogeneous nucleation, oblate spheroidal-shaped Cu_2_O nanoparticles formed, which cover the majority of the TiO_2_ coating surface, and have an average size of 326 ± 89 nm. Under the Cu_2_O particles, the porous structure of the TiO_2_ coating is clearly visible (Figure 4c) (additional SEM images can be seen in Figure S6). Elemental maps were recorded by EDS measurements: an example SEM image and its elemental map are shown in Figure 4e,f, while additional elemental maps can be seen in Figure S7. The EDS measurements confirmed that copper (Cu Lα1,2), oxygen (O Kα1), and titanium (Ti Lα1,2) elements were present in the sample in a homogeneous distribution throughout the investigated sample area. Silicon (Si Kα1) from the substrate and carbon (C Kα1,2) from organic pollutants were also observed. The average atomic content of the sample was found to be 41.8% Cu, 33.5% O, 6.7% Ti, 3.4% Si, and 14.6% C. The copper/oxygen ratio was calculated by also taking into account the presence of TiO_2_: the oxygen amount needed for TiO_2_ was subtracted from the total oxygen content before determining the Cu/O quotient. The determined Cu/O ratio was 2.1, which confirms that the particles are made of Cu_2_O, without the presence of Cu or CuO.

**Figure 4:**
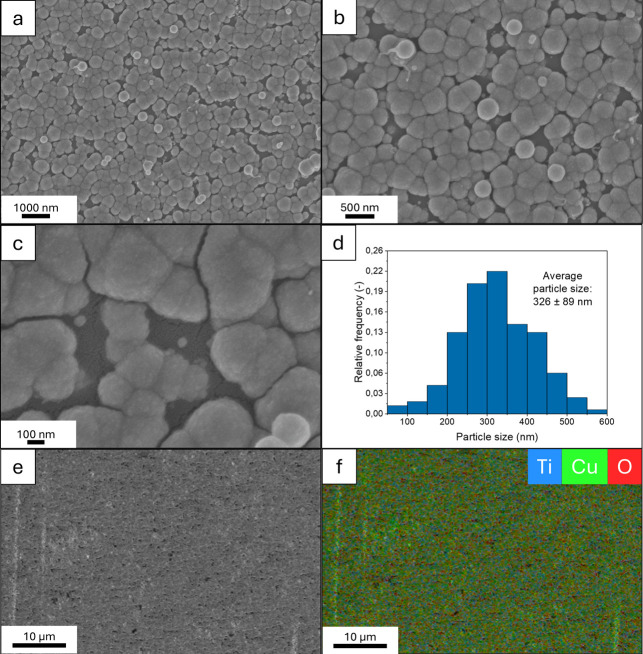
FE-SEM images (a–c), size distribution diagram of Cu_2_O particles (d); chosen SEM image (e) and its elemental map determined by EDS (f) of TiO_2_/Cu_2_O coatings (prepared with a 60 min reaction time). Average Cu_2_O particle sizes were calculated from diameter values of 200 particles measured on the micrographs.

The properties of the layer formed by Cu_2_O particles (prepared with a 60 min reaction time) were further characterized with AFM images (Figure 5). Almost the entire surface of the TiO_2_ coating is covered by a single layer of sphere-like Cu_2_O particles, which shows good agreement with the SEM images (Figure 4). The single-layer formation of the layer is not perfect, since within the investigated area, some pin-holes can be seen where the TiO_2_ surface is not completely covered, and at a few locations there are Cu_2_O particles visible, which formed on the top of the first layer of particles (also shown in a cross-sectional diagram in Figure S8).

**Figure 5:**
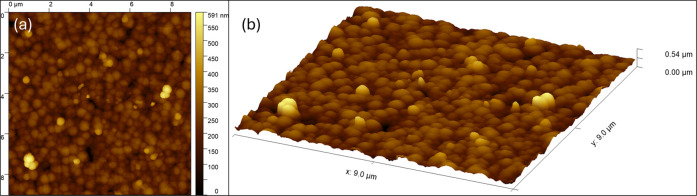
2D (a) and 3D (b) AFM images of TiO_2_/Cu_2_O coatings (prepared with a 60 min reaction time) on a 9 × 9 μm scan area.

Transmittance spectra of the samples before and after Cu_2_O particle deposition can be seen in Figure 6. The decreased transmittance that can be observed after particle deposition is caused by the absorption and light scattering of the Cu_2_O nanoparticles. Lower transmittance was observed with increasing reaction time, which indicates that the surface coverage can be controlled with the immersion time of the titania samples in the reaction mixture.

**Figure 6:**
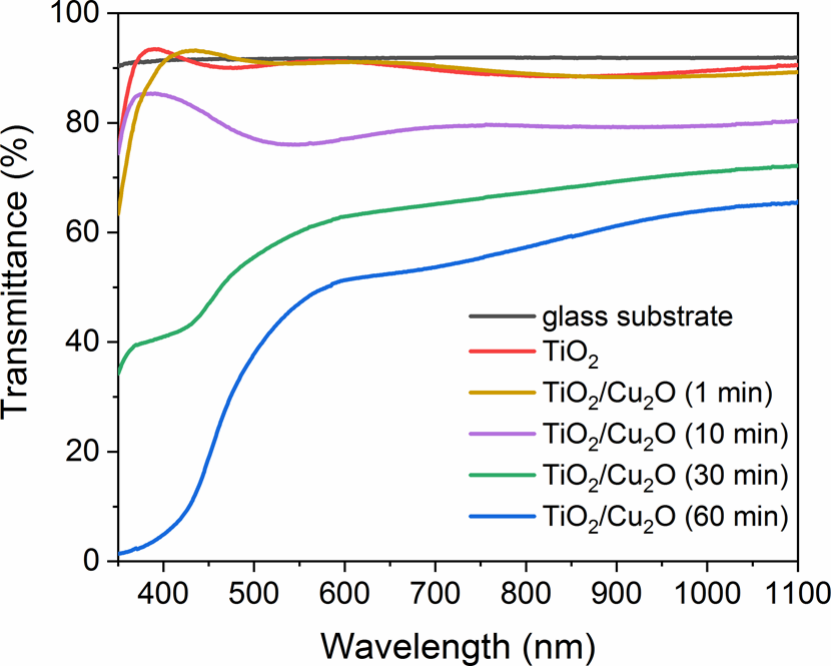
Transmittance spectra of coatings before and after Cu_2_O particle deposition.

Thin film optical model fitting was carried out on the transmittance spectra of the reference samples (coatings without Cu_2_O particles); the results can be seen in Table S1. The mesoporous TiO_2_ coatings were found to have a thickness of 122 nm and a porosity of 49%.

Spectroscopic ellipsometry was used to determine the layer thickness and porosity values of the TiO_2_/Cu_2_O composite coatings (prepared with a 60 min reaction time), and the band gap energy values of TiO_2_ and Cu_2_O. The mesoporous titania layer was found to have a thickness of 125 nm and a porosity of 51%, which is in good agreement with the results obtained from the thin film optical fitting of the transmittance spectra (see detailed results in Supporting Information Figure S9 and Table S2). The layer of the deposited Cu_2_O particles was found to have a thickness of 330 nm and a porosity of 20% (meaning that 20% of the layer is made of air-filled voids in between the Cu_2_O particles; the Cu_2_O particles are nonporous). Additionally, by taking into account the FE-SEM images of the coating, which show particles with an average size of 326 nm (see Figure 4c–d), it can be said that using this deposition method, on average the thickness suggests that a mostly single layer of Cu_2_O particles with a sphere-like (oblate spheroidal) shape formed on the titania surface. This is not true for the entire sample surface, as some FE-SEM images (see Figure S6) clearly show a multilayered structure at some locations of the surface. The band gap energy values of TiO_2_ and TiO_2_/Cu_2_O composite coatings (prepared with a 60 min reaction time) were determined to be 3.43 and 2.49 eV, respectively, based on Tauc plots obtained from the ellipsometry measurements (see Figure S10). The band gap energy of the Cu_2_O layer was found to be 2.14 eV based on the multilayer ellipsometric optical model, which includes the Tauc–Lorentz model with a band gap parameter. The Cu_2_O particle deposition onto the TiO_2_ surface caused significant bend banding and resulted in a 0.94 eV decrease of the band gap. The results show good agreement with literature values: Pavan et al.^46^ prepared TiO_2_/Cu_2_O coatings with spray pyrolysis and found that the band gap energy of the composite was ∼2.48 eV.

### 3.2 Photocatalytic Activity of the Samples

The photoactivity of the samples was studied in dye photodegradation tests, under UV or visible light. The absorbance spectra of the dye solutions were measured as a function of irradiation time, and the maximum absorbance values of the peak at 465 nm (attributed to the absorption of methyl orange^47^) were determined. Photoactivity of different samples was compared by plotting *A*/*A*_0_ as a function of the irradiation time, where *A*_0_ is the initial value of maximum absorbance (obtained after reaching the adsorption equilibrium, before the start of irradiation), and *A* is the maximum absorbance value after a given irradiation time. (See additional details in the Supporting Information, in Figures S11 and S12.) Reference samples (coatings without photoactive materials Cu_2_O and/or TiO_2_; photoactive coatings kept in darkness) were used in order to separate absorbance decrease occurring due to other degradation processes (e.g., reactions with H_2_O_2_, heat effects, etc.) from photocatalytic degradation.

Figure 7a shows the photocatalytic activity of the TiO_2_/Cu_2_O coatings under UV light irradiation. The unmodified TiO_2_ coating was used as a reference sample since TiO_2_ also shows photoactivity under UV light. The photodegradation test indicates that the samples that were kept in the Cu_2_O reaction mixture for 1 and 10 min degraded more methyl orange dye than the unmodified TiO_2_, while longer reaction times resulted in less dye degradation. This implies that Cu_2_O particles are able to enhance the photoactivity of TiO_2_ coating under UV light, if the surface coverage is adjusted suitably. The presence of Cu_2_O particles on the TiO_2_ surface can improve photocatalytic degradation: the recombination of the photoexcited charge carriers produced by the TiO_2_ is inhibited due to the presence of Cu_2_O; furthermore, Cu_2_O particles can also themselves absorb UV photons and take part in generating charge carriers. However, covering the surface of the TiO_2_ with Cu_2_O particles can also have a disadvantageous effect: the presence of the particles decreases the free TiO_2_ surface area; some of the inner mesoporous structure of the layer becomes no longer accessible to the dye molecules, which also decreases the overall specific surface area of the sample. Longer reaction times (30 and 60 min) led to high surface coverage by Cu_2_O particles, suppressing the availability of the TiO_2_ active sites for the adsorption of the dye and ultimately leading to lower photoactivity compared to the reference TiO_2_ coating that contains no Cu_2_O particles.

**Figure 7:**
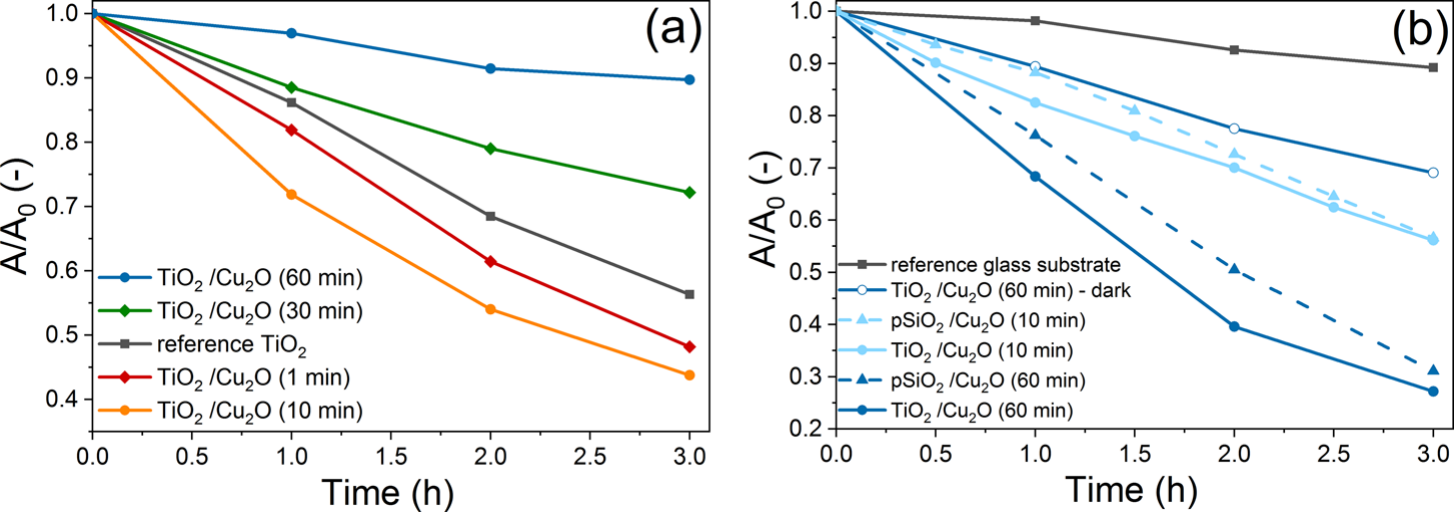
Photocatalytic activity of the TiO_2_/Cu_2_O samples in methyl orange dye solution under (a) UV and (b) visible light irradiation.

The sample found to be most effective under UV light (prepared with a 10 min reaction time) and the sample containing the most Cu_2_O particles (prepared with a 60 min reaction time) were selected for further photoactivity tests under visible light (see Figure 7b). Several reference samples were also studied to evaluate the effectivity of Cu_2_O particle photoactivity and the role of TiO_2_ in promoting the photocatalytic process. Glass substrate was used as a reference to measure the absorbance decrease occurring due to nonphotodegradation processes, such as dye molecules reacting with the H_2_O_2_ cocatalyst. Mesoporous SiO_2_/Cu_2_O reference samples were used (silica is inert under visible light irradiation), prepared identically to TiO_2_/Cu_2_O coatings, in order to study the effect of the presence of TiO_2_. A TiO_2_/Cu_2_O (60 min) coating was kept in dark under identical conditions (placed in continuously stirred dye solutions for the same time intervals) to evaluate the degradation processes that can occur without irradiation, of which there are several possible mechanisms: First, the oxygen molecules adsorbed on the Cu_2_O surface can oxidize to reactive superoxide radicals, which can perform a redox reaction with the methyl orange molecule.^48^ Also, the Cu_2_O particles can undergo cyclic redox reactions with the H_2_O_2_ molecules, whereby the Cu^2+^ ion is reduced to the Cu^+^ ion by reacting with a H_2_O_2_ molecule and then oxidized to the Cu^2+^ ion by another H_2_O_2_ molecule, while at the same time reactive hydroxyl and hydroperoxyl radicals are formed from the H_2_O_2_ molecules, which can perform a redox reaction with the methyl orange molecule.^49^

Compared to the reference glass substrate and the sample kept in dark, all TiO_2_/Cu_2_O coatings showed a higher degree of dye degradation under visible light illumination, with the samples prepared with a reaction time of 60 min showing the highest photoactivity. This is likely due to the much higher number of Cu_2_O nanoparticles formed on the surface, compared to the samples prepared with a 10 min reaction time. In comparison with the previous results under UV irradiation, where both TiO_2_ and Cu_2_O can be excited, TiO_2_ is not photoactive under visible light, and only the Cu_2_O is responsible for photon absorption and generation of charge carriers. The samples that contained TiO_2_ showed a slightly faster dye degradation than the SiO_2_/Cu_2_O reference coatings, which indicates that the presence of the TiO_2_ was also beneficial in visible light, since the separation of charge carriers is enhanced by the interaction between Cu_2_O and TiO_2_: the electrons can travel from the conduction band of the Cu_2_O particles to the conduction band of TiO_2_, while holes move from the valence band of TiO_2_ to the valence band of Cu_2_O particles.^9^

Comparing the measurements in visible and UV light illumination, in visible light it was beneficial to have a large amount of Cu_2_O particles on the surface, whereas in UV light the almost full coverage of the TiO_2_ surface was disadvantageous. This is due to the different photocatalytic mechanisms under different illuminations, since under visible light the primary photoactive material is the Cu_2_O, while under UV light the TiO_2_ is also photoactive, and it is important to have enough accessible area for the dye molecules on its surface. There might be other factors that affect the photodegradation, such as thickness and surface roughness of the Cu_2_O layer and the size distribution of the nanoparticles. By increasing the amount of Cu_2_O nanoparticles on the surface of the titania, the photoactivity under visible light may become more effective; however, with the loss of free active sites on the titania surface, the photoactivity under UV light decreases, as confirmed by our results. An optimal surface coverage must exist, where the photocatalysis is the most effective under sunlight, which would be most useful for real-life applications, and it contains both visible and UV light. It is important to note that the increased amount of Cu_2_O nanoparticles might also affect the specific surface area of the overall coating system: depending on the nanoparticle sizes and on the thickness and the structure of the formed nanoparticle layer, the surface area available to the dye molecules may increase or decrease, which strongly affects the overall efficiency. Further investigations are needed to uncover the relationship between these properties and to develop photocatalysts with the optimal parameters.

It is also important to note that after immersing the samples in aqueous dye solutions for 4 h during these tests, the transmittance spectra of the TiO_2_/Cu_2_O coatings only showed a negligible change (see Figure S13), suggesting that the particles remained on the surface, and were not removed by immersion in dye solutions for 4 h. As an additional stability test, the same photodegradation test (3 h illumination in dye solutions) was carried out again on the same samples that are presented in Figure 7. The photodegradation test was repeated after 4 years of storage, and remarkably, the coating samples still showed measurable photoactivity in comparison to the reference glass substrate, even after being immersed in an aqueous solution for 4 h, followed by the notably long 4 years of storage time, and the repeated 4 h immersion (see Figure S14). These results show that the Cu_2_O particles formed via heterogeneous nucleation have a strong adhesion to the titania surface, resulting in stable composite coatings.

## 4 Conclusion

TiO_2_/Cu_2_O composite coatings were prepared using heterogeneous nucleation for deposition of Cu_2_O nanoparticles onto the mesoporous titania surface (made with sol–gel dip-coating method, with anatase crystal structure, a thickness of 122 nm, and a porosity of 49%). It was found that Cu_2_O particles (average particle size: 326 nm) with an oblate spheroidal shape, cubic crystal structure, and a band gap energy of 2.14 eV formed on the titania surface, without the presence of Cu or CuO particles. The surface coverage could be controlled by the immersion time of the titania coating in the reaction mixture.

Dye photodegradation tests under UV and visible light were carried out to study the photoactivity of the samples. It was found that the TiO_2_/Cu_2_O composite coatings were photoactive under both UV and visible light and showed a more efficient dye degradation than the reference samples (coatings without photocatalysts, or kept in the dark). Using the method presented in this paper, high surface coverage of crystalline Cu_2_O particles could be achieved in a simple, one-step deposition process, resulting in TiO_2_/Cu_2_O composite coatings with good photoactivity under visible light. The surface coverage can be easily controlled by immersion time, making it possible to achieve a suitable photoactive property for various applications; e.g., by adjusting the surface coverage, coatings with promising photoactivity under both UV and vis irradiation can be prepared.

## Supplementary Material



## Notes

Schematic of the sample preparation and photos of the samples (Figure S1); Raman spectra of glass substrate (Figure S2); TiO_2_/Cu_2_O composite coating prepared with a 30 min reaction time (Figure S3); results of XPS measurements (Figures S4 and S5); FE-SEM images and elemental maps (Figures S6 and S7); AFM image and cross-sectional diagram (Figure S8); results of optical model fitting based on measured UV–vis spectroscopy (Table S1); spectroscopic ellipsometry (Table S2); schematic model of the Cu_2_O coating (Figure S9); Tauc plots (Figure S10); additional photocatalysis data (Figures S11–S13); and results of the repeated photodegradation test (Figure S14) (PDF)
