# Supplementary material for: Titania-Supported Photocatalytic Coatings of Cu2O Nanoparticles Synthesized via Heterogeneous Nucleation
Source: ACS Omega. 2026 Jan 20;11(4):5374–83. doi: 10.1021/acsomega.5c08505 (PMC12878506; doi:10.1021/acsomega.5c08505)
Supplement: Supplementary file 1 [file ao5c08505_si_001.pdf]

Supporting Information

Titania-Supported Photocatalytic Coatings of Cu_2O Nanoparticles Synthesized via Heterogeneous Nucleation

Petra Demény[a], *Borbála Tegze*[a,*], *Bálint Fodor*[b], *Pál Maák*[c], *János Madarász*[d], *Zsombor Pap*[b],

Dániel Zámbó[e], *Tamás Igricz*[f], *Adél Sarolta Rácz*[e], *Norbert Nagy*[e], *Zoltán Hórvölgyi*[a,*]

[a] Department of Physical Chemistry and Materials Science, Faculty of Chemical Technology and Biotechnology, Budapest University of Technology and Economics, Műegyetem rkp. 3., H-1111 Budapest, Hungary;
[*]Email: tegze.borbala@vbk.bme.hu, horvolgyi.zoltan@vbk.bme.hu;
Phone number: +36 1 463 1111/5772; +36 1 463 2911

[b] Semilab Semiconductor Physics Laboratory Co. Ltd., Prielle Kornélia u. 2, H-1117 Budapest, Hungary

[c] Department of Atomic Physics, Faculty of Natural Sciences, Budapest University of Technology and Economics, Műegyetem rkp. 3., H-1111 Budapest, Hungary

[d] Department of Inorganic and Analytical Chemistry, Faculty of Chemical Technology and Biotechnology, Budapest University of Technology and Economics, Műegyetem rkp. 3., H-1111 Budapest, Hungary

[e] Institute for Technical Physics and Materials Science, HUN-REN Centre for Energy Research, Konkoly-Thege M. út 29-33., H-1121 Budapest, Hungary

[f] Department of Organic Chemistry and Technology, Faculty of Chemical Technology and Biotechnology, Budapest University of Technology and Economics, Műegyetem rkp. 3., H-1111

Preparation of TiO_2/Cu_2O coating samples

Figure S1 illustrates the experimental steps carried out during the preparation of TiO_2/Cu_2O coating samples. The photos in Figure S1 (lower right) show that increasing immersion time lead to a drastic change in the colour of the sample, due to the increased Cu_2O layer thickness.

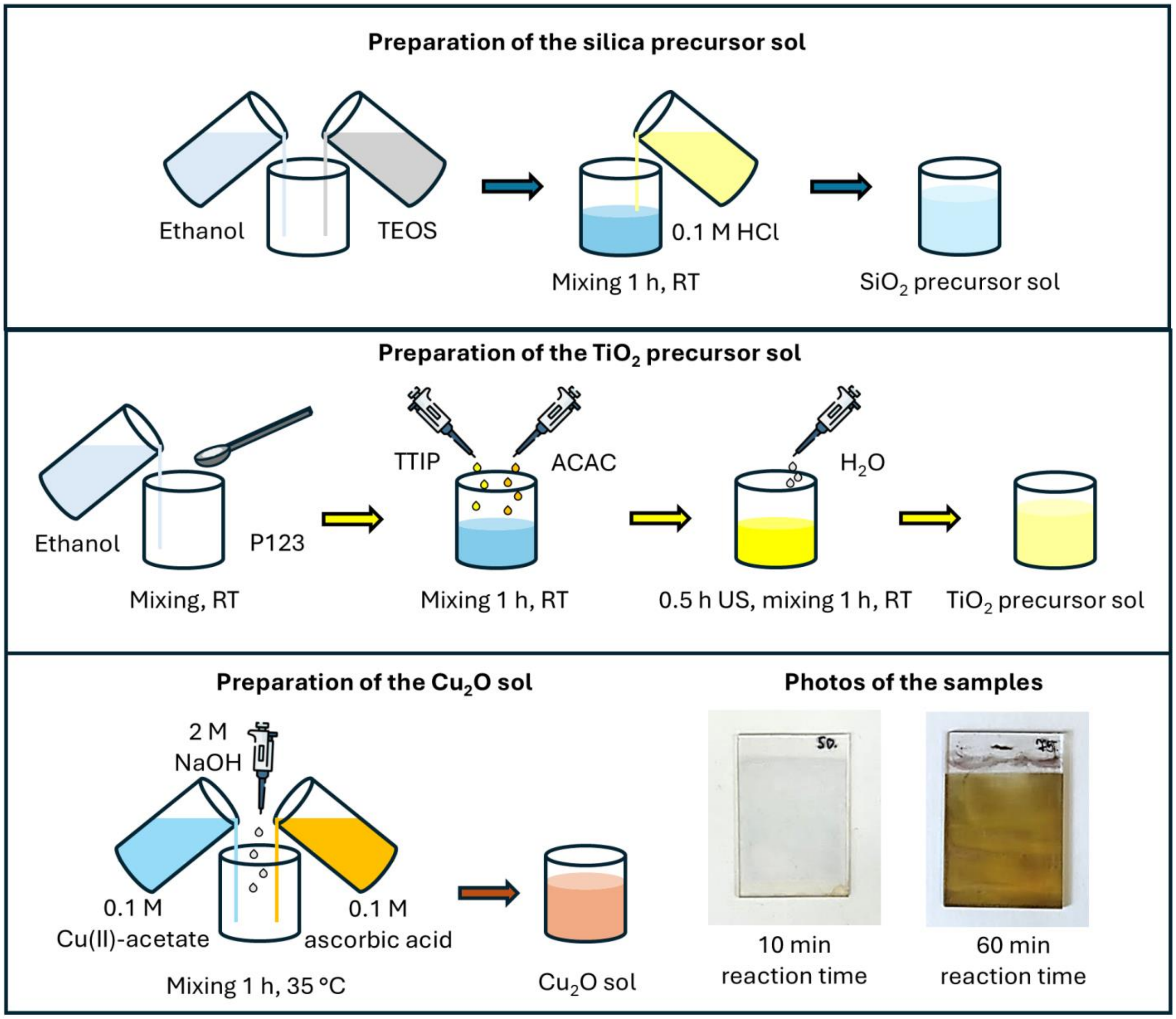

Figure S1. Sample preparation: Experimental steps of silica and TiO_2 precursor sol synthesis; preparation of the Cu_2O sol; and photos of the TiO_2/Cu_2O coating samples prepared with 10 min (left) and 60 min (right) immersion time

Raman spectroscopy

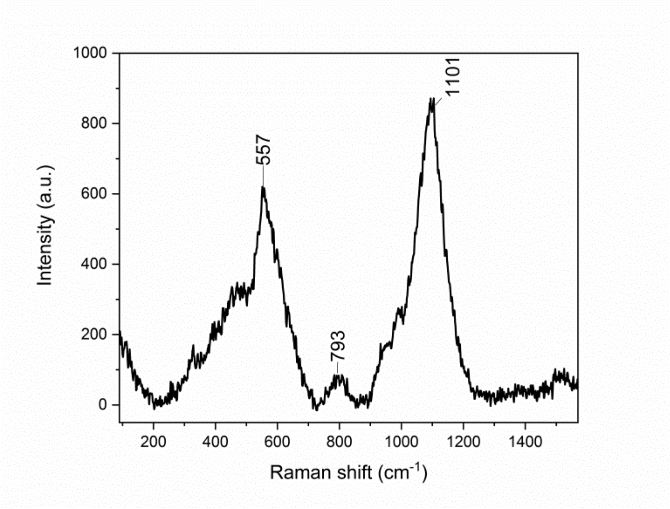

Figure S2. Raman spectrum of glass substrate

The characteristic bands of the Cu_2O can be observed in Raman spectrum of the TiO_2/Cu_2O sample prepared on glass substrate with 30 min reaction time (Figure S3).

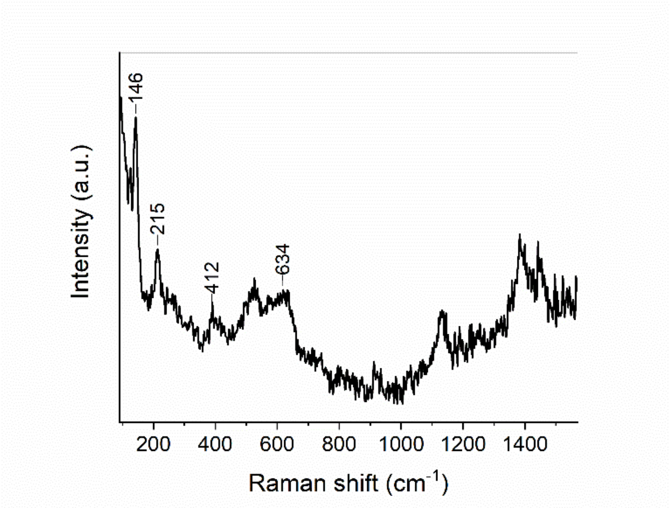

Figure S3. Raman spectrum of TiO_2/Cu_2O sample prepared on glass substrate with 30 min reaction time

The Raman spectra of the samples prepared with 1 min and 10 min reaction time were also obtained, but the characteristic bands were not observable, due to the very small amount of particles on the surface.

XPS measurement

X-ray photoelectron spectroscopy (XPS) measurement was carried out on a TiO_2/Cu_2O sample (prepared with 60 min reaction time on silicon substrate). Transition metal oxides are known to be reduced especially under monoatomic argon sputtering [*Sakai, Y. et al. (2012) 10.1002/sia.4843*] therefore a cluster argon ion source with low energy cluster size ratio (4 keV, cluster size 2000, angle of incidence 45° respect to the surface normal) was used to decrease the surface contamination of the sample. Thermo Scientific Avantage Software was used for data evaluation. After reducing the backgrounds, the atomic concentrations were obtained by applying the sensitivity factor library (Althermo1). The sample showed a very low level of charging (0.1-0.2. eV), therefore, due to uncertainties of using adventitious carbon as binding energy calibration [*Greczynski, G. et al (2021) 10.1016/j.apsusc.2020.148599*] the data are presented as received.

Silicon (2p), carbon (1s), copper (LMM), copper (2p), titanium (2p), oxygen (1s), sulfur (2p) and nitrogen (1s) high-resolution spectra were measured. The XPS Survey spectrum can be seen on Figure S4.

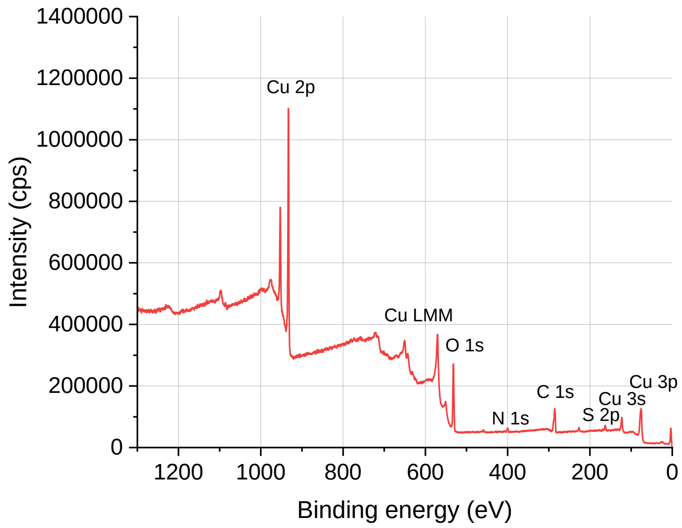

Figure S4. XPS spectrum of TiO_2/Cu_2O coating (prepared with 60 min reaction time on silicon substrate)

The atomic composition of the sample was determined to be 37.8% C 1s, 23.8% Cu 2p, 30.2% O 1s, 0.9% Ti 2p, 4.0% S 2p and 3.3% N 1s. There was no detectable amount of Si 2p. The high-resolution spectrum of the Cu 2p showed two peaks at 952.6 ± 0.1 eV and 932.5 ± 0.1 eV, which corresponds to Cu 2p 1/2 and Cu 2p 3/2, respectively. The main component at 932.5 ± 0.1 eV is characteristic for Cu (I) oxide and Cu (0) [*Biesinger, M. C. et al (2017) 10.1002/sia.6239*]. For identifying this chemical state, the Cu $L_3M_{4,5}M_{4,5}$ Auger peak and the modified Auger parameter were used. The calculated Auger parameter value was found to be 1849.1 ± 0.1 eV, which is characteristic for Cu (I) oxide, indicating that metallic copper is not present in the sample.

The Cu 2p XPS and Cu $L_3M_{4,5}M_{4,5}$ Auger spectra can be seen on Figure S5.

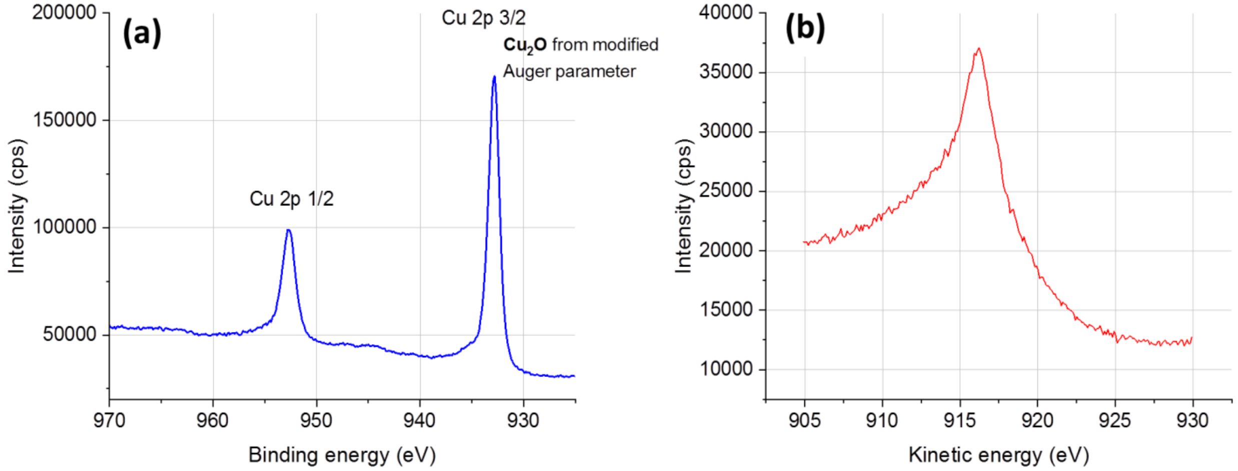

Figure S5. Cu 2p XPS spectrum (a) and Cu $L_3M_{4,5}M_{4,5}$ Auger spectrum (b) of TiO_2/Cu_2O coating (prepared with 60 min reaction time on silicon substrate)

FE-SEM images and EDS elemental maps

Figure S6. shows the FE-SEM image of the TiO_2/Cu_2O coating sample (prepared with 60 min reaction time). It can be observed, that at some locations, the Cu_2O particles form multiple layers on the surface.

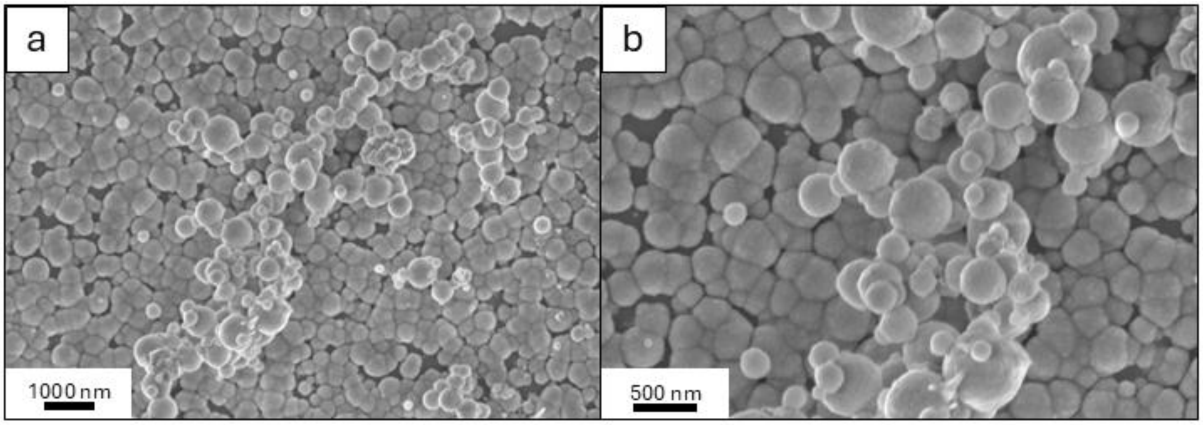

Figure S6. Additional FE-SEM images (a-b) of TiO_2/Cu_2O coatings (prepared with 60 min reaction time)

Elemental maps

Elemental maps of the TiO_2/Cu_2O sample (prepared with 60 min reaction time on silicon substrate) were determined by SEM-EDS. The brightness of each color corresponds to the intensity of the element: the brighter the color, the more intense the presence of the element.

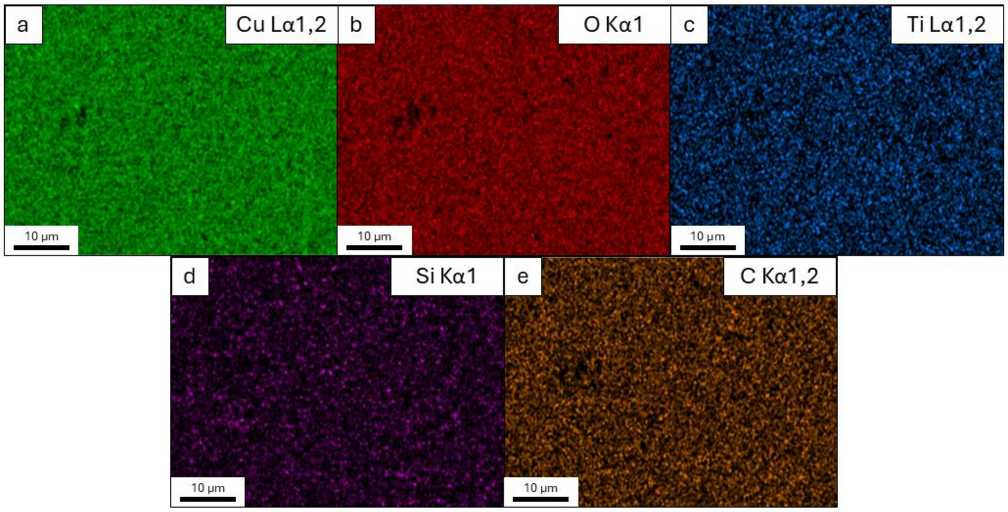

Figure S7. Elemental maps of copper (a), oxygen (b), titanium (c), silicon (d) and carbon (e) elements of TiO_2/Cu_2O sample (prepared with 60 min reaction time on silicon substrate)

AFM

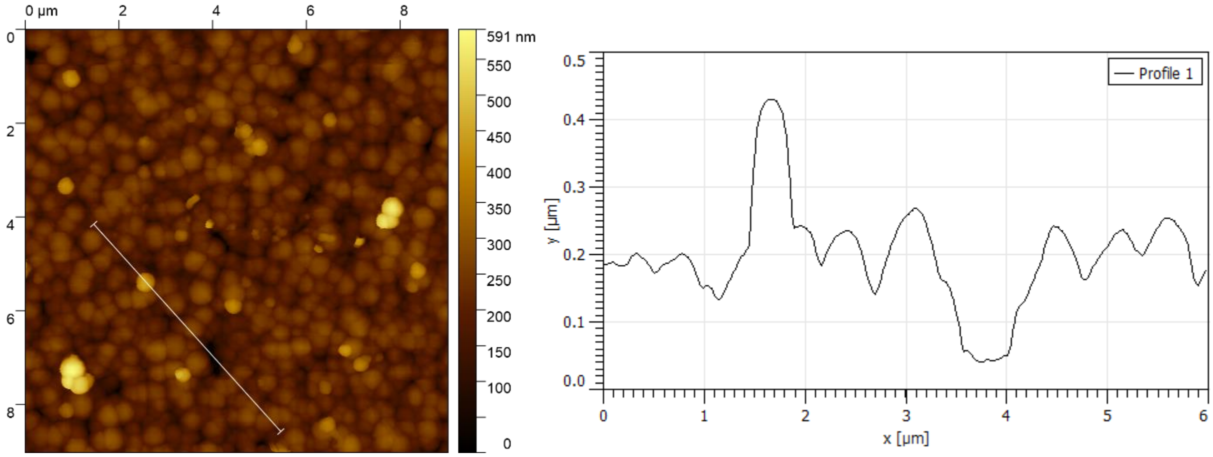

Figure S8. Additional AFM image (left) and a corresponding cross-section diagram (right) of TiO_2/Cu_2O coatings (prepared with 60 min reaction time)

UV-Vis spectroscopy

Results of thin film optical model fitting based on the measured transmittance spectra:

Table S1: Effective refractive index, layer thickness and porosity values of the mesoporous TiO_2 coatings, the compact silica barrier layers and the mesoporous silica reference coatings.

Sample	Effective refractive index [-]	Layer thickness [nm]	Porosity [%]
TiO_2	1.534 ± 0.006	122 ± 2	49 ± 1
compact silica	1.449 ± 0.002	208 ± 3	-
mesoporous silica	1.323 ± 0.003	145 ± 3	27 ± 1

Spectroscopic ellipsometry

The spectra measured by spectroscopic ellipsometry were analyzed layer-by-layer to determine refractive index, layer thickness and porosity values of the component layers of TiO_2/Cu_2O coatings. The compact silica layer was analyzed with the Sellmeier model, the titania and the Cu_2O coatings were studied with the Tauc-Lorentz model. The model of the Cu_2O coating was built up of 3 layers as shown in Figure S9. The refractive index and the layer thickness values were determined for each layer of the Cu_2O model (Table S2). The porosity was calculated for each layer and the average of it was defined as the porosity of the Cu_2O coating (20%). The sum of each layer thickness was determined as the Cu_2O coating's thickness (330 nm).

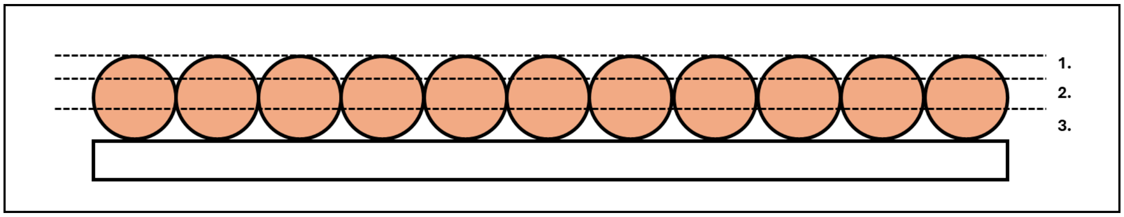

Figure S9. The three layers used in the model for analysis of the coating made of Cu_2O nanoparticles

Table S2: Effective refractive index, layer thickness and porosity values of the compact silica barrier layer, the mesoporous TiO_2 coating and the 3 layers of the Cu_2O coating.

Layers of the TiO_2/Cu_2O sample	Effective refractive index [-]	Layer thickness [nm]	Porosity [%]
compact silica	1.453	213	-
TiO_2	1.526	125	51
Cu_2O layer 1.	1.925	33	28
Cu_2O layer 2.	2.291	133	11
Cu_2O layer 3.	2.028	164	23

Tauc plots

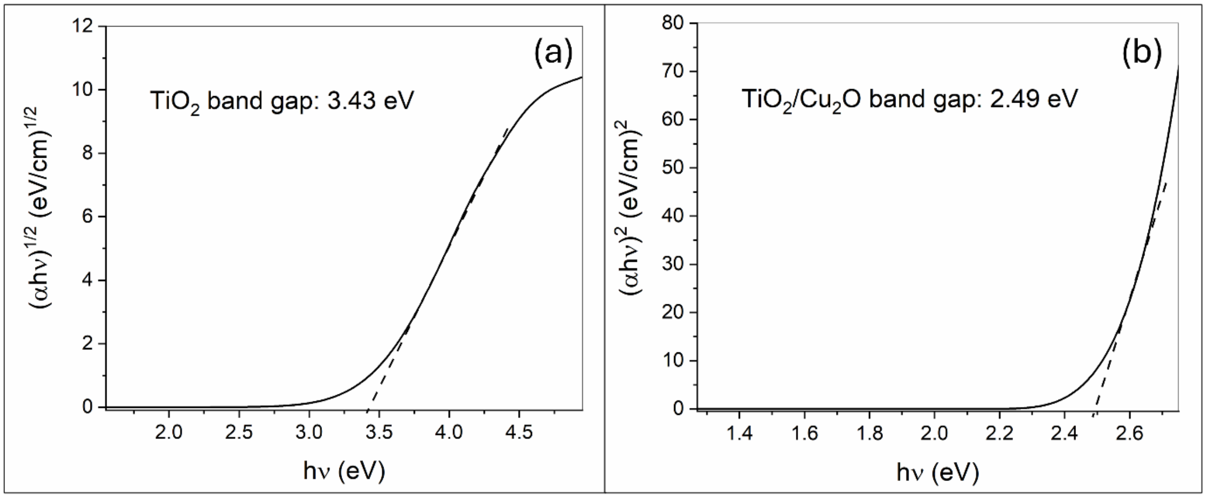

Figure S10. Tauc plots of the TiO_2 coating (a) and the TiO_2/Cu_2O composite coating (b)

Photocatalysis tests

Before starting irradiation by UV or visible light, the samples were immersed in the dye solutions, and kept in darkness for 1 hour, in order to measure the absorbance decrease due to possible adsorption of the dye molecules on the surface of the sample ("adsorption step"). The measured absorbance spectra change during this adsorption step can be seen in Figure S11 (measured on a TiO_2/Cu_2O sample prepared using 10 min reaction time). An example of the measured absorbance spectra change during irradiation can be seen in Figure S12 (under visible light, measured on a TiO_2/Cu_2O sample prepared using 10 min reaction time).

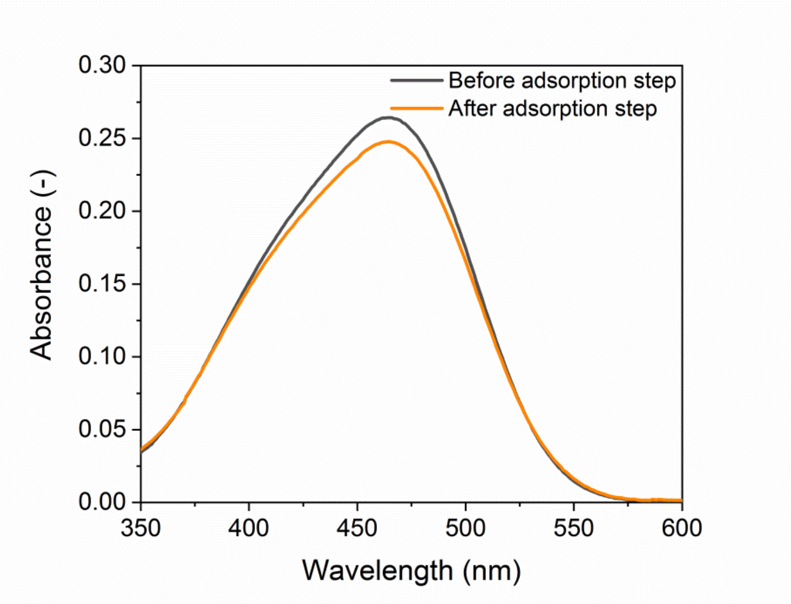

Figure S11. The change of the methyl orange dye solution's absorption spectra during the adsorption step

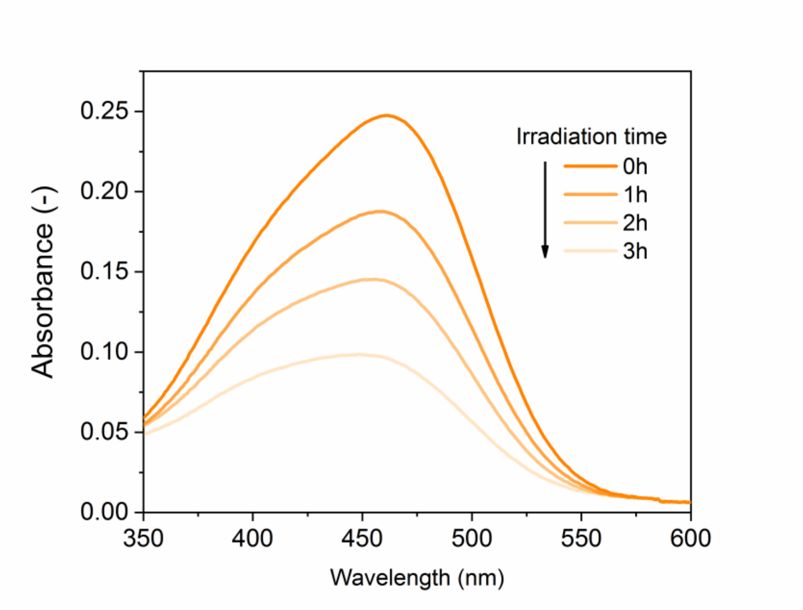

Figure S12. Absorbance spectra of the methyl orange dye solution during the photocatalysis test

Transmittance spectra of the coatings before and after immersion in dye solutions for 4 hours can be seen in Figure S13.

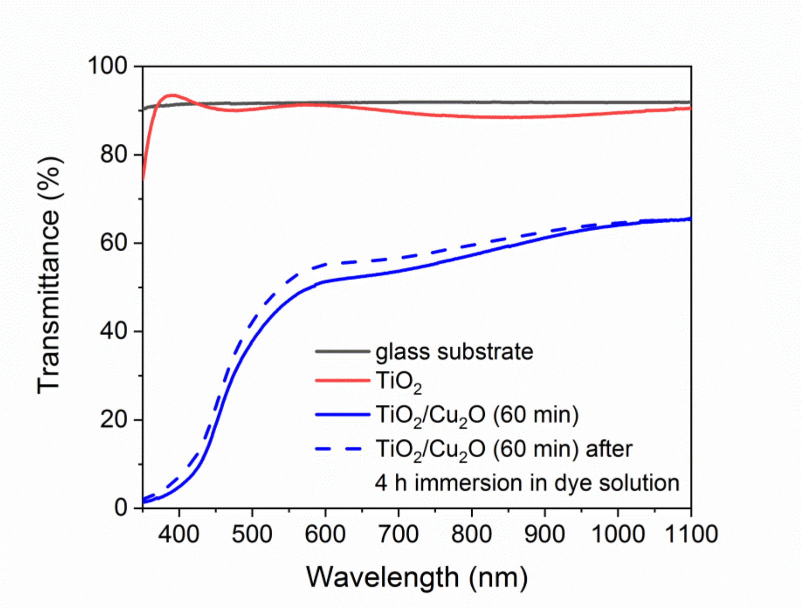

Figure S13. Transmittance spectra of the TiO_2/Cu_2O sample (prepared with 60 min reaction time) before and after the photocatalysis test was carried out

Repeated photodegradation tests

Additional repeated photodegradation tests under both UV and visible light were carried out on samples that were previously illuminated for 3 h in methyl orange solutions, and then were stored for 4 years. After 4 years of storage another 4 h dye photodegradation test (1 h immersion in darkness, followed by 3 h illumination) was carried out using the same method and parameters as before. (The original photodegradation test reported in our manuscript was carried out within 1 month of preparing the coating samples.)

The results can be seen on Figure S14, comparing the performance with the one measured 4 years prior:

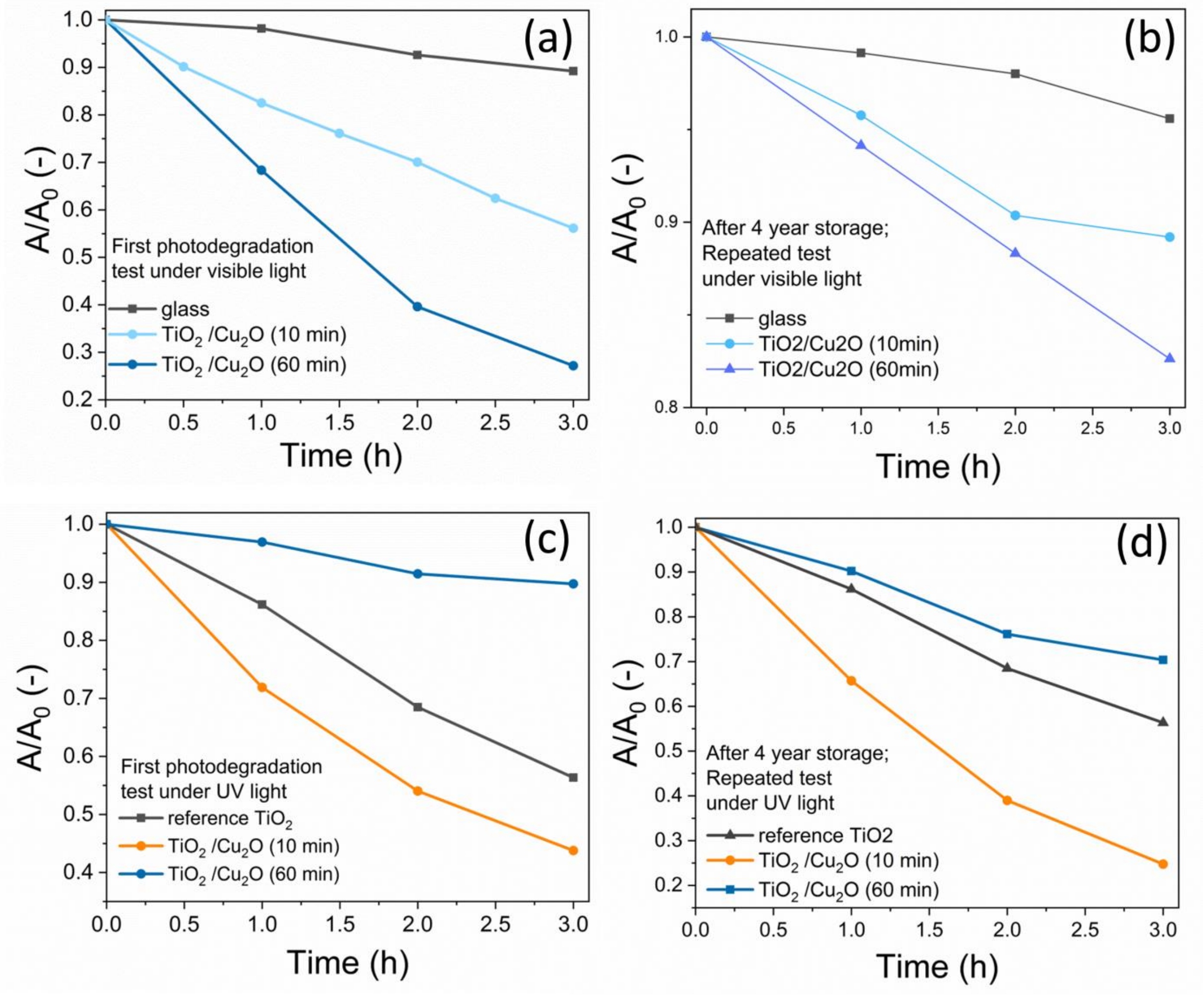

Figure S14. The results of photodegradation tests of the TiO_2/Cu_2O samples in methyl orange dye solution under visible light irradiation carried out within 1 month of preparing the samples (a), and repeated on the same samples after 4 years of storage (b).

Under visible light, the photoactivity of the samples significantly decreased: originally the dye absorbance peak maximum value decreased by ~40% and ~70% during the 3 h visible light illumination, in the case of the TiO_2/Cu_2O sample prepared with 10 and 60 min reaction time, respectively; compared to the ~10% (10 min reaction time) and ~20% (60 min reaction time) decrease achieved after the repeated photodegradation test. However, it is remarkable that the coating samples still showed measurable

photoactivity in comparison to the reference glass substrate, even after being immersed in an aqueous solution for 4 hours, followed by 4 years of storage.

Under UV light, the photoactivity of the samples remained unchanged, or it even increased compared to the results from 4 years earlier: the reference TiO_2 coating (without Cu_2O particles) showed originally ~40% decrease in the dye absorbance peak maximum value during the 3 h UV light illumination, and in the repeated photodegradation test a very similar value was achieved, the decreased was still ~40%. In comparison, the decrease was originally ~60% in the case of the TiO_2/Cu_2O sample prepared with 10 min reaction time, while ~70% was measured in the repeated test. Similarly, the decrease was originally ~10% in the case of the TiO_2/Cu_2O sample prepared with 60 min reaction time, while ~30% was measured in the repeated test. In both cases, after 4-year storage a slightly higher photoactivity of the TiO_2/Cu_2O samples could be measured in the repeated test. This is most likely due to some of the Cu_2O particles being removed from the surface, as the results suggest that under UV light a low surface coverage by Cu_2O particles on the titania surface is preferred. Still, not all Cu_2O particles were removed from the surface, since the photoactivity enhancing effect of the presence of the Cu_2O particles (compared to the reference TiO_2 coatings) is clearly measurable in the case of the TiO_2/Cu_2O samples prepared with 10 min reaction time.

These results demonstrate that the Cu_2O nanoparticles deposited *via* heterogeneous nucleation have good adhesion to the titania surface, as they were not completely removed even by soaking for long times in an aqueous solution, nor did the coating degrade or separate from the substrate during the notably long storage time of 4 years.
